# Melatonin promotes ripening and improves quality of tomato fruit during postharvest life

**DOI:** 10.1093/jxb/eru332

**Published:** 2014-08-21

**Authors:** Qianqian Sun, Na Zhang, Jinfang Wang, Haijun Zhang, Dianbo Li, Jin Shi, Ren Li, Sarah Weeda, Bing Zhao, Shuxin Ren, Yang-Dong Guo

**Affiliations:** ^1^College of Agriculture and Biotechnology, China Agricultural University, Beijing, China; ^2^School of Agriculture, Virginia State University, Petersburg, USA

**Keywords:** Ethylene, gene expression, melatonin, postharvest, ripening, tomato.

## Abstract

In this study, the effect of melatonin on the postharvest ripening and quality improvement of tomato fruit was carried out. The tomatoes were immersed in exogenous melatonin for 2h, and then the related physiological indicators and the expression of genes during post-harvest life were evaluated. Compared with control check (CK), the 50 µM melatonin treatment significantly increased lycopene levels by 5.8-fold. Meanwhile, the key genes involved in fruit colour development, including phytoene synthase1 (*PSY1*) and carotenoid isomerase (*CRTISO*), showed a 2-fold increase in expression levels. The rate of water loss from tomato fruit also increased 8.3%, and the expression of aquaporin genes, such as *SlPIP12Q*, *SlPIPQ*, *SlPIP21Q*, and *SlPIP22*, was up-regulated 2- to 3-fold under 50 µM melatonin treatment. In addition, 50 µM melatonin treatment enhanced fruit softening, increased water-soluble pectin by 22.5%, and decreased protopectin by 19.5%. The expression of the cell wall modifying proteins polygalacturonase (PG), pectin esterase1 (PE1), β-galactosidase (TBG4), and expansin1 (Exp1) was up-regulated under 50 µM melatonin treatment. Melatonin increased ethylene production by 27.1%, accelerated the climacteric phase, and influenced the ethylene signalling pathway. Alteration of ethylene production correlated with altered 1-aminocyclopropane-1-carboxylic acid (ACC) synthase (ACS4) expression. The expression of ethylene signal transduction-related genes such as *NR*, *SlETR4*, *SlEIL1*, *SlEIL3*, and *SlERF2*, was enhanced by 50 µM melatonin. The effect of melatonin on ethylene biosynthesis, ethylene perception, and ethylene signalling may contribute to fruit ripening and quality improvement in tomato. This research may promote the application of melatonin on postharvest ripening and quality improvement of tomato fruit as well as other horticultural productions in the future.

## Summary

Tomato fruits were treated by exogenous melatonin. The effect of melatonin on ethylene biosynthesis, ethylene perception, and ethylene signalling may contribute to fruit ripening and quality improvement in tomato.

## Introduction

Fruit ripening is a highly coordinated, genetically programmed, irreversible phenomenon involving a series of physiological, biochemical, and organoleptic changes that lead to changes in colour, texture, flavour, aroma, and nutritional status (Prasanna *et al.*, 2007; Rugkong *et al.*, 2011). Tomato fruit ripening is accompanied by changes in colour from green to red, softening, and increased levels of compounds that contribute to flavour and aroma, such as organic acids, sugars and volatiles. The colour change is due to the unmasking of previously present pigments by degradation of chlorophyll coupled with the synthesis of different types of anthocyanins and the accumulation of carotenoids such as β-carotene, xanthophyll esters, xanthophylls, and lycopene. Indeed, lycopene is considered a major carotenoid in tomato that provides red colour (Ronen *et al.*, 1999). Softening is thought to be the result of cell wall disassembly, decreased cell adhesion (Vicente *et al.*, 2007; Lunn *et al.*, 2013), and the cumulative effects of reducing cellular turgor pressure (Shackel *et al.*, 1991). The changes in cell wall structure are accompanied by a solubilization of pectins and depolymerization of hemicellulosic polysaccharides.

In addition to the structural matrices of the cell wall, another important contributor to texture and fruit firmness is cellular turgor. Cellular turgor is governed by the water status within fruit and the relative water distribution within the cell and in the cell wall (Seymour *et al.*, 2013). Water transport across biological membranes is facilitated by water channel proteins called aquaporins (Chrispeels and Maurel, 1994). Aquaporin proteins during ripening regulate the passage of water from the symplast to the apoplast, which would presumably also affect water movement out of the cell (Seymour *et al.*, 2013).

Flavour is defined as the combination of taste and odour. Aroma of the ripe fruit is attributed to the production of a complex mixture of volatile compounds such as hexanal, myrcene, and ocimene, and degradation of bitter principles, tannins, flavonoids, and related compounds (Prasanna *et al.*, 2007). The majority of plant volatiles on a quantitative and qualitative basis originate from saturated and unsaturated fatty acids (Schwab *et al.*, 2008). Hexanal is formed by lipid oxidation of unsaturated fatty acids on the maceration of fruit, which is critical to ripe aroma and tomato-like flavour (Baldwin *et al.*, 1998; Alexander and Grierson, 2002). The taste development is due to a general increase in sweetness, which is the consequence of increased gluconeogenesis, hydrolysis of polysaccharides, especially starch, decreased acidity, and accumulation of sugars and organic acids resulting in an excellent sugar/acid blend (Prasanna *et al.*, 2007).

Tomato (*Solanum lycopersicum* L.) is one of the most important horticultural crops. It has long served as a model system for studying fleshy fruit development and ripening owing to its relatively small genome, ease of genetic manipulation, well‐characterized developmental mutants, and relatively short life cycle. Tomato belongs to the climacteric class of fruits, which includes banana, apple, and pear. Most climacteric fruits show increased ethylene production at or just before the onset of ripening and require ethylene to complete the process (Alexander and Grierson, 2002). Therefore, ethylene synthesis, perception, and signalling are very important events for fruit ripening. The pathway of ethylene biosynthesis has been determined (Adams and Yang, 1979). The key enzyme is 1-aminocyclopropane-1-carboxylic acid (ACC) synthase (ACS), which regulates the production of ACC from *S*-adenosylmethionine (SAM) (Alexander and Grierson, 2002; Prasanna *et al.*, 2007). ACC oxidase (ACO) is an important enzyme for the control of ethylene production. Ethylene perception is by the target cells through receptors (ETRs), which act as negative regulators of the ethylene response pathway. The signal transduction cascade involves both positive and negative regulators (CTR, EIN2, EIN3 etc.) and regulation of target gene expression by transcription factors such as ethylene response factors (ERFs) (Olmedo *et al.*, 2006; Bapat *et al.*, 2010).

Melatonin, or *N*-acetyl-5-methoxytryptamine, is a hormone found in animals, plants, and microbes (Manchester *et al.*, 2000; Burkhardt *et al.*, 2001; Paredes *et al.*, 2009; Posmyk and Janas, 2009). Besides its function as synchronizer of the biological clock, melatonin is also a powerful free-radical scavenger and wide-spectrum antioxidant (Tan *et al.*, 1993). Melatonin was initially discovered in plants by two groups in 1995 (Dubbels *et al.*, 1995; Hattori *et al.*, 1995). Since then, it has been detected in the roots, leaves, flowers, fruits, and seeds of a considerable variety of plant species (Cao *et al.*, 2006; Okazaki and Ezura, 2009; Park, 2011; Lei *et al.*, 2013; Zhao *et al.*, 2013). Melatonin concentration varies among plant organs depending on the given physiological and environmental conditions. Its highest level was found in flowers and seeds, which may be related to their high sensitivity to environmental stresses, e.g. UV irradiation (Posmyk and Janas, 2009). However, little is known about its physiological function in plants. Nocturnal increases of melatonin were detected in *Chenopodium rubrum*, suggesting that melatonin in plants may have functions analogous to those in animals (Kolar *et al.*, 2002). Melatonin also showed antioxidative activities in plants as observed in animals (Rodriguez *et al.*, 2004). Melatonin plays regulatory roles in plant metabolism (Wang *et al.*, 2014), acts as a growth-regulatory signal similar to auxin, delays flower induction (Kolar *et al.*, 2003), slows root formation (Hernandez-Ruiz *et al.*, 2004), and promotes adventitious and lateral root regeneration (Zhang *et al.*, 2013, 2014).

As a healthy ingredient contained in the diet, many fruits, including apple, cherry, banana, strawberry, pineapple, grape, and tomato, provide natural melatonin (Reiter *et al.*, 2005; Sae-Teaw *et al.*, 2013). Melatonin was first detected in wild tomato species *Solanum pimpinellifolium* (currant tomato) in 1995. It was also reported that melatonin accumulates in mature tomato fruits and seeds, and the melatonin content in the pericarp of tomato fruit increased from mature green stage to red stage (Okazaki and Ezura, 2009; Feng *et al.*, 2014). However, the role of melatonin in tomato fruit ripening is not well understood.

In this study, we focused on the effect of melatonin on the postharvest ripening of tomato fruit, and revealed the mechanism of melatonin on ethylene biosynthesis, lycopene synthesis, cell wall structure, and water loss. This research may promote the application of melatonin on postharvest ripening and quality improvement of tomato fruit as well as other horticultural productions in the future.

## Materials and methods

### Regents

All chemicals used in experiments were of analytical grade. Melatonin (*N*-acetyl-5-methoxytryptamine) was purchased from Sigma-Aldrich (St. Louis, MO, USA). All other reagents were purchased from Sinopharm Chemical Reagent Beijing Co., Ltd, China.

### Plant material

The following experiments were conducted at China Agricultural University, Beijing (39.9_N 116.3_E). The tomato plant used in this study, variety “Bmei”, is a cherry tomato. Fruits were collected at the green stage of maturity with homogeneous size and randomly grouped into three lots (100 fruits per lot) for treatment in triplicate.

### Fruit treatment

The tomato fruits were rinsed briefly in water before treatment to avoid soil contamination. After that, the tomatoes were immersed in solution for 2h. The five solutions made for treatments were: control (distilled water), M1 (1 µM melatonin), M50 (50 µM melatonin), M100 (100 µM melatonin), and M500 (500 µM melatonin). Following immersion, the fruits were dried for 15min at room temperature (RT). The tomato fruits were then stored for 25 days at 15 °C and 80% relative humidity. Three independent trials were carried out. At each sampling date, fruit were used for measurements of ethylene production, colour, and firmness, and then the pericarp tissues were frozen with liquid nitrogen, powdered, and stored at –80 °C until further use.

### Melatonin extraction and analysis

A total of 4g tomatoes were homogenized with 10ml methanol, then ultra-sonicated (80 Hz) for 35min at 45 °C. After centrifugation at 10 000g at 4 °C for 15min twice, the supernatants were collected and dried under nitrogen gas. Samples were dissolved in 2ml 5% methanol and transferred to a C18 solid phase extraction (SPE) cartridge (ProElut^TM^, DIKMA, China) for the purification of melatonin. Melatonin was extracted and analysed as described previously (Zhao *et al.*, 2013). The mobile phase was a mixture of acetonitrile: 50 mmol l^–1^ Na_2_HPO_4_/H_3_PO_4_ buffer pH 4.5 (15:85), which was delivered at a flow rate of 1.0ml min^–1^. The injection volume of the extract was 10 µl. Melatonin was detected using excitation and emission wavelengths at 280 and 348nm, respectively. Samples were determined in triplicate.

### Determination of ethylene production

Ethylene production of the tomato fruit was measured by enclosing three fruits in 5 L air-tight containers for 2h at 20 °C, withdrawing 1ml of the headspace gas with a syringe, and injecting it into a gas chromatograph (model Agilent, 6890N, USA) fitted with a flame ionization detector and an activated alumina column.

### Fruit firmness and water loss measurement

Fruit firmness was measured at the furthest two points apart on the equator of each fruit (i.e. equidistant between the top and bottom of each fruit) with a Texturometer (Model GY-B, Xingke, Jilin, China) fitted with a 2mm plunger.

The percent weight loss of tomato fruit samples was calculated by taking the difference between initial weight and final weight divided by initial weight. Data was recorded for twenty biological replicates each in three independent sets.

### Soluble sugar measurement

To measure the content of soluble sugars, 0.5g of tomato tissue was homogenized with 5ml of 95% ethanol. One hundred µl of alcoholic extract was mixed with 3ml anthrone (150mg anthrone, 50ml of 72% sulphuric acid, W/W) 50. The samples were boiled for 10min. The light absorption of the samples was measured at 630nm using a 2800UV/VIS model spectrophotometer. Contents of soluble sugar were determined using sucrose standard.

### Pectin measurement

Tomato tissue (0.5g) derived from 4 fruits, free of placental tissue and seeds, was homogenized with 25ml 95% ethanol, and boiled for exactly 30min. After cooling at RT, the reaction was centrifuged at 8000g for 15min, the supernatant was removed, and the pellet suspended in 25ml 95% ethanol and boiled for 30min. After repeating 3–5 times, the pellet was suspended in 20ml distilled water, and incubated in a 50 °C water bath for 30min. The mixture was then centrifuged at 8000g for 15min, and the supernatant containing the water-soluble pectin was transferred to a 100ml volumetric flask. The pellet was dissolved with 25ml 0.5mol l^–1^ H_2_SO_4_ and boiled for 1h. Following centrifugation at 8000g for 15min, the supernatant containing the proto-pectin was transferred to a 100ml volumetric flask.

The pectin content was determined by mixing 1ml of collected pectin with 6ml of concentrated hydrochloric acid. The reaction was boiled for 20min, cooled in tap water, followed by the addition of 0.2ml 1.5g l^–1^ carbazole and a 30min incubation in the dark at RT. The absorbance at 530nm was measured against reagent blanks and pectin content was calculated based on a galacturonic acid standard curve.

### Lycopene content measurement

Lycopene content was measured at 17 d after melatonin treatment as described in Fish *et al*. (Fish *et al.*, 2002) with modifications. Partially thawed tomato pericarp tissue (5g) derived from 4 fruits, free of placental tissue and seeds, and 50ml of hexane–acetone–ethanol (2:1:1, v/v) were homogenized for 1min wrapped in aluminum foil. After homogenization, 15ml of water was added and the samples vortexed for 10 s. Following phase separation on ice, lycopene concentration was determined by measuring the absorbance of the organic phase (hexane) at 503nm. All the procedures were performed under dim light. Lycopene content was calculated using the molar extinction coefficient of 17.2 l mol^−1^m^−1^ and expressed on a fresh weight basis as mg kg^−1^. Three independent samples derived from four fruits at each measurement interval were used for lycopene measurement.

### Extraction of volatiles and gas chromatography-mass spectrometry analysis

The aroma components were analysed by SPME/ GC-MS according to Liu *et al*. (Liu *et al.*, 2011). Pericarp tissue (20g) was obtained from tomato fruits, immediately frozen in liquid nitrogen, and stored at –80 °C until extraction. The MS was operated using an EI ion source, with a temperature of 170 °C, electron energy 70eV, and photomultiplier tube voltage of 350V. A 2-µl aliquot of the pooled organic phases (extracted as described above), was directly injected into the gas chromatograph-mass spectrometer for volatile analysis; at least two extractions for each sample were performed.

The ion source and the transfer line were set to 200 °C and 260 °C, respectively. Volatile compounds were separated on a column (30 m×0.25mm inside diameter, 0.25 µm film). The column temperatures were programmed as follows: 60 °C for 2min, raised to 220 °C at 8 °C min^−1^, then held for 20min. The injector temperature was 250 °C. Helium was the carrier gas at 1ml min^−1^ in the splitless mode. Electron impact mass spectra were recorded in the 30–550 amu range with a scanning speed of 0.5 scan s^−1^.

### RNA Extraction, reverse transcription-PCR and real-time quantitative PCR assay

Total RNA was extracted from the fruits using TRIzol reagent according to the manufacturer’s protocol (Invitrogen, Burlington, ON, Canada). The quality and quantity of all RNA samples were assessed by agarose gel electrophoresis and spectrophotometry (Thermo NanoDrop^**TM**^ 2000c, USA). First-strand cDNA was reverse transcribed from 2 µg of total RNA using the PrimeScript^**TM**^ RT reagent kit (Takara, Japan) according to the manufacturer’s instructions.

Primers used for real-time PCR, designed using Primer 5 software, are listed in Table S1. Specificity of each primer to its corresponding gene was checked using the BLASTN program of the NCBI. Real-time PCR was performed using the SYBR Premix Ex Taq™ kit (TaKaRa, Japan). Reactions contained 1 µl of primer mix, 2 µl cDNA template, 10 µl SYBR Premix Ex Taq™ (2×) mix, and 7 µl water for a total volume of 20 µl. Reactions were carried out under the following conditions: 95 °C/30 s (1 cycle); 95 °C/10 s, 60 °C/34 s (40 cycles), using ABI Prism 7500 Sequence Detection System and Software (PE Applied Biosystems, USA). To normalize sample variance, Tomato Sl-Actin-51 (accession No. Q96483) gene was used as endogenous control. To determine relative fold differences for each sample, the Ct value of genes was calculated relative to a calibrator using the formula 2^–ΔΔCt^. At least two to three independent RNA isolations were used for cDNA synthesis and each cDNA sample was subjected to real-time PCR analysis in triplicate.

### Statistical analysis

Statistical analysis was performed by one-way analysis of variance (ANOVA). Means were compared by the Fisher’s LSD test at a significance level of 0.05.

## Results

### Melatonin enhanced pigment accumulation of tomato fruit

**Fig. 1.**
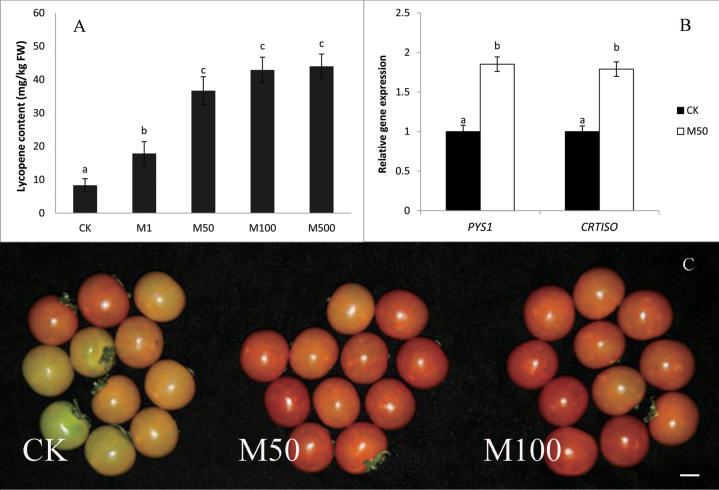
Melatonin’s effect on tomato pigment accumulation.CK: fruits pre-treated with water; M50: fruits pre-treated with 50 µmol l^–1^ melatonin; M50: fruits pre-treated with 50 µmol l^–1^ melatonin. (A) Lycopene content at 17 d after treatment. (B) Real-time PCR analysis of PSY1 and CRTISO expression levels in fruit at 17d after treatment. Vertical bars at each time point represent the least significant difference (LSD) test when significant at *P*=0.05. (C) The colour of tomato at 17 d after treatment. The scale bar indicates 1cm.

Lycopene, one of the main carotenoids in tomato, is mainly responsible for the red colour and varies with different ripening stages. To test the effect of melatonin on tomato fruit ripening during postharvest, preliminary experiments were carried out to determine the appropriate concentrations of melatonin. In this study, we found that lycopene accumulated significantly in melatonin-treated fruits (Fig. 1C) at 17 d after melatonin treatment (DAT), compared with the control (CK). A 5.1-fold increase was observed with 50 µM melatonin treatment (36.7mg per g FW), and there were no significant changes among 50 µM, 100 µM, and 500 µM melatonin (Fig. 1A).

**Fig. 2.**
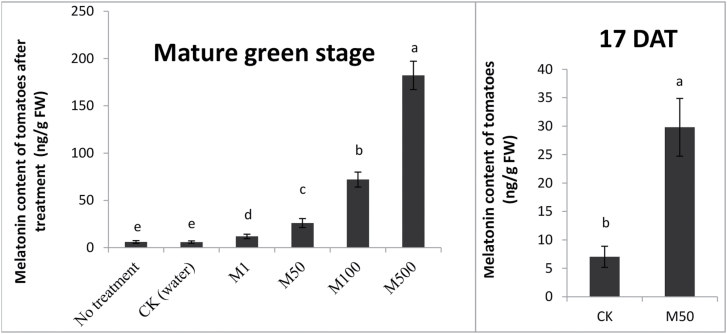
Melatonin content in tomato fruits after treatment. Tomatoes were treated without (water) or with melatonin in concentrations: 1, 50, 100, and 500 µM. The melatonin content of tomatoes after treatment at (A): mature green stage and (B): 17 DAT. Vertical bars at each time point represent the LSD when significant at *P*=0.05.

To know how much melatonin was absorbed by the tomatoes, we measured melatonin contents of the fruits. Compared with CK (water treatment), the melatonin contents of melatonin-treated mature green tomatoes were increased significantly. The highest melatonin contents were 182.2ng per g FW in M500, and gradually decreased as exogenous melatonin concentration declined. The MT contents were 72.0, 25.9, 11.8, and 5.9ng per g FW in MT100, MT50, MT1, and CK, respectively (Fig. 2A). Previous studies reported the occurrence of melatonin in various tomatoes; the melatonin contents ranged from 0.6–114.5ng per g FW (Sturtz *et al.*, 2011; Feng *et al.*, 2014). Thus, melatonin application at 50 µM seemed to be the most effective as it produced the highest level of lycopene and the lowest residue level of melatonin. Therefore, 50 µM melatonin was used in this study.

To better understand the effects of exogenously applied melatonin on melatonin contents in tomato fruits, we also measured melatonin concentrations of the fruits at 17 DAT (day after melatonin treatment) (Fig. 2B). The melatonin contents at 17 DAT in both CK and M50 were higher than those of the corresponding mature green fruits; the melatonin concentrations were 7.0 and 29.8ng per g FW in CK and M50, respectively.

To further understand the effect of melatonin on the lycopene accumulation in tomato fruit, we analysed the expression of *SlPSY1* and *SlCRTISO* genes, which are involved in lycopene biosynthesis. In general, both of these genes were up-regulated about 2-fold in the melatonin-treated fruits (Fig. 1B). The enhanced expression of lycopene synthesis genes following melatonin treatment was consistent with increased lycopene accumulation and colour change observed in M50-fruits. The result suggests that melatonin may have a role in mediating lycopene biosynthesis-related genes to alter lycopene concentration in tomato fruit, and finally affect the colour change of tomato fruit.

### Melatonin softened and changed cell wall structure

**Fig. 3.**
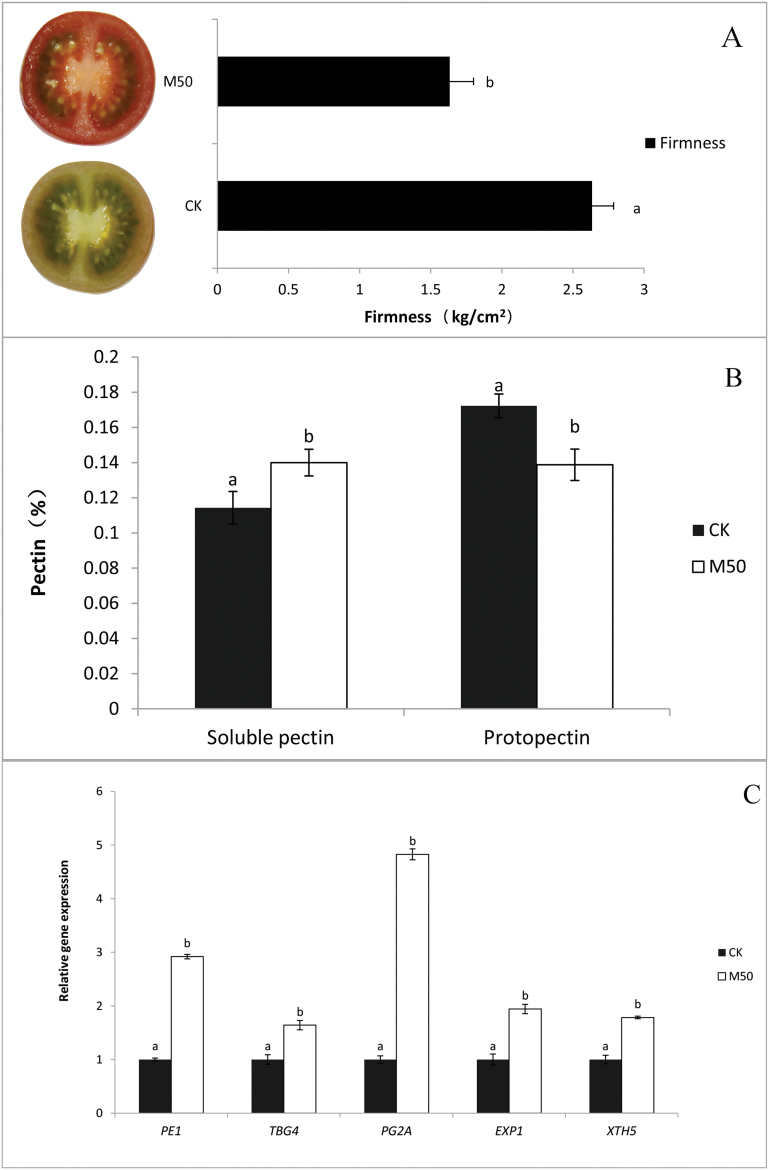
Melatonin affects softening and firmness of tomato fruit. CK: fruits pre-treated with water; M50: fruits pre-treated with 50 µmol l^–1^ melatonin. (A) Firmness. (B) Pectin level. (C) Real-time PCR analysis of cell wall structure-related gene expression in tomato fruits at 17 d after treatment. Vertical bars at each time point represent the LSD when significant at P=0.05. (This figure is available in colour at *JXB* online.)

Fruit softening was measured using a Texturometer to test fruit pericarp firmness. The data showed that control tomatoes were substantially firmer than melatonin-treated fruits at 17 DAT. The firmness decreased by 38.0% in M50-fruits compared with CK. (Fig. 3A)

To reveal whether the cell wall structure changed, we also measured the content of pectin, which is a structural heteropolysaccharide contained in the primary cell walls of plants. We found that the content of proto-pectin was reduced by 19.5%, whereas the soluble pectin content increased 22.5% in M50-fruits (Fig. 3B).

To fully understand how melatonin changes cell wall structure in tomato fruit, five cell wall structure-related genes were investigated. The expression of cell wall structure-related genes (*SlPE1*, *SlTBG4*, *SlPG2a*, *SlExp1* and *SlXTH5*) following 50 µM melatonin treatment was generally higher than untreated controls. The expression of the *SlPG2a* gene, which is involved in the hydrolysis of pectin, was sharply increased by almost 5-fold in M50-fruits when compared with CK-fruits (Fig. 3C). The result that *SlPG2a* expression was up-regulated in the melatonin-treated fruits was consistent with reduced pectin levels in these tomato fruits.

At 17 DAT, 50 µM melatonin-treated fruits were getting softer than control fruit (Fig. 3A), and the expression of cell wall structure-related genes (*SlPE1*, *SlTBG4*, *SlPG2a*, *SlExp1*, and *SlXTH5*) was up-regulated by 50 µM melatonin treatment (Fig. 3C) was consistent with reduced pectin level in the M50 fruit. It indicates that melatonin may lead to fruit softening by up-regulating cell wall structure-related genes, especially the *SlPG2a* gene, which is responsible for much of the depolymerization of de-esterified pectic homogalacturonans (Hadfield and Bennett, 1998; Brummell and Harpster, 2001).

Texture of fruit not only affects consumer preference, but also has a significant impact on shelf life and transportability. Shelf life is the recommended maximum time for which products can be stored, during which the defined quality of a specified proportion of the goods remains acceptable under expected (or specified) conditions of distribution, storage, and display. High quality tomato has a firm, turgid appearance, uniform and shiny colour, without signs of mechanical injuries, shrivelling or decay. Mature-green tomatoes have a storage life of 1–3 weeks in normal atmosphere storage (NA) at 13–21 °C (Burg, 2004). In the present study, both melatonin-treated and control groups had a similar shelf life and a good merchantability at 21 DAT.

### Melatonin treatments increased rate of water loss and aquaporin expression

**Fig. 4.**
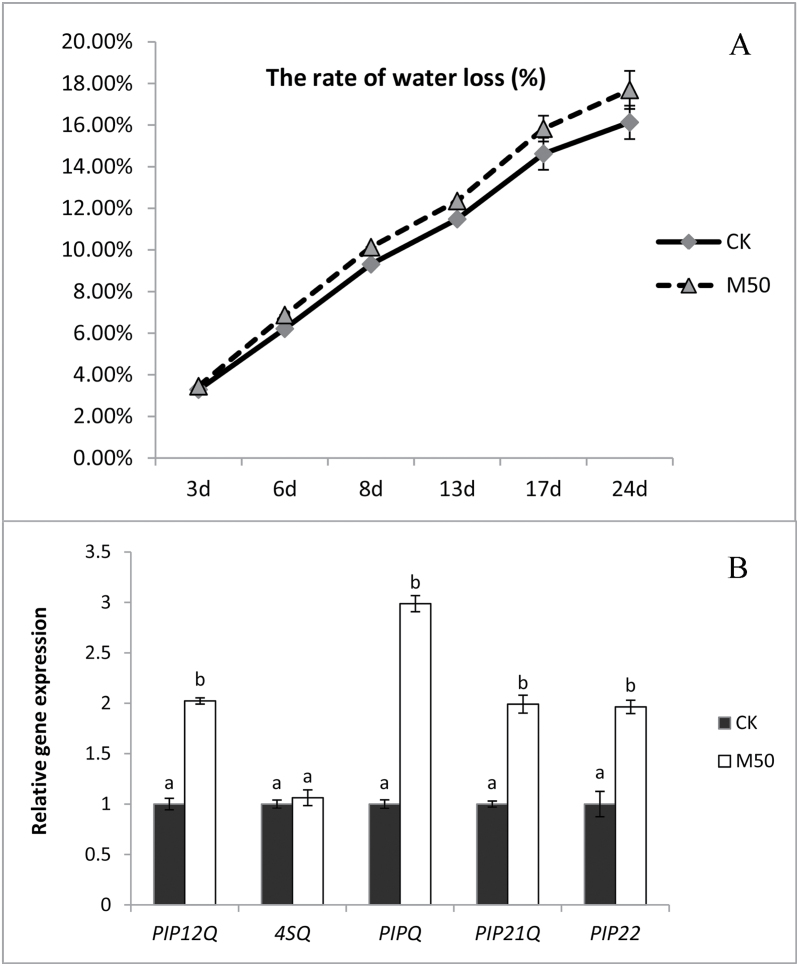
Melatonin has an effect on water loss in tomato fruit. CK: sample pre-treated with water; M50: sample pre-treated with 50 µmol l^–1^ melatonin. (A) The rate of water loss of tomato during postharvest storage. (B) Real-time PCR analysis of aquaporin genes expression in tomato fruits at 17 d after treatment. Vertical bars at each time point represent the LSD when significant at P=0.05.

Initially, no significant changes in fruits weight loss (%) were observed in control fruits and melatonin-treated fruits. However, as ripening proceeded, the weight loss significantly (*P*<0.5) increased by 8.3% and 9.7% at 17 DAT and 24 DAT, respectively, in melatonin-treated fruits (Fig. 4A).

Furthermore, we analysed the effect of melatonin treatment on the expression of five aquaporin genes, which are considered to be factors that influence water loss, in tomato fruits at 17 DAT. Our results indicate that, compared with CK, *SlPIP12*, *SlPIP21* and *SlPIP22* expression levels had a 2-fold increase, and *SlPIPQ* had a 3-fold increase in M50-fruits, whereas *Sl4SQ* did not change significantly (Fig. 4B). These results suggest that melatonin may affect water loss in fruit by altering the expression of some aquaporin genes.

### Melatonin increased ethylene production and influenced on ethylene signalling pathway

Ethylene production was determined during fruit storage. As shown in Fig. 4A, ethylene production was highest in melatonin-treated fruits at 17 DAT, whereas the climacteric peak of CK appeared at 19 DAT. The climacteric peak of 50 µM melatonin-treated fruit was higher, and the production of ethylene was increased by 27.1% in the peak when compared with CK. Our data indicate that melatonin enhanced ethylene production, and affected the timing of the climacteric peak.

**Fig. 5.**
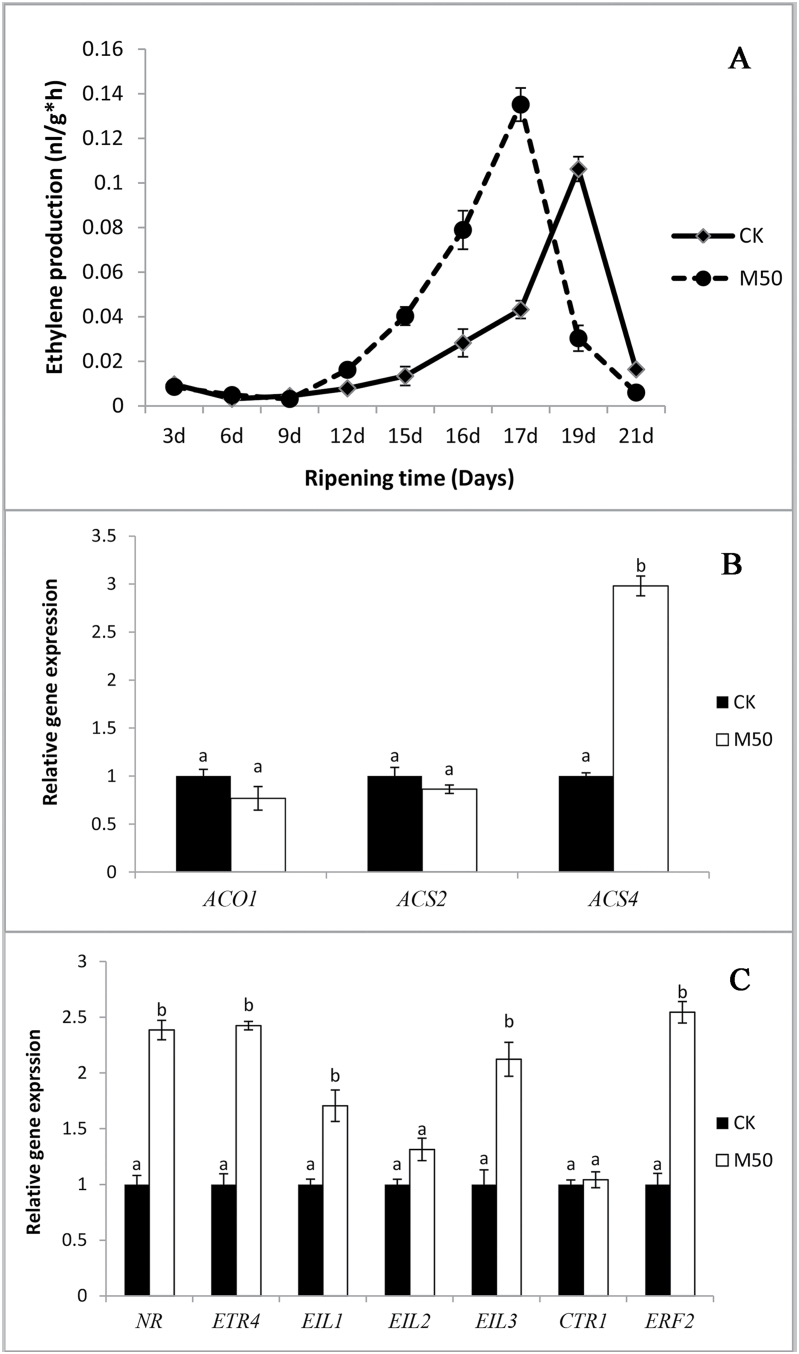
Melatonin has an effect on ethylene production, signalling pathway, and relative gene expression. CK: sample pre-treated with water; M50: sample pre-treated with 50 µmol l^–1^ melatonin. (A) Ethylene production of tomato fruits during postharvest storage. (B) Real-time PCR analysis of ethylene biosynthesis-related genes expression in tomato fruits at 17 d after treatment. (C) Real-time PCR analysis of ethylene signalling-related gene expression in tomato fruits at 17 d after treatment. Vertical bars at each time point represent the LSD when significant at *P*=0.05.

To determine whether the expression of ethylene biosynthesis-related genes was consistent with the above results, we analysed the gene expression of *SlACS2*, *SlACS4*, and *SlACO1*, which are responsible for autocatalytic ethylene production during fruit ripening (Klee and Giovannoni, 2011). Compared with CK, *SlACS4* was up-regulated 3-fold in M50, whereas *SlACS2* and *SlACO1* showed no significant changes in response to melatonin treatment (Fig. 5B). These results revealed that melatonin might increase ethylene production by enhancing *SlACS4* expression.

To better understand the role of melatonin in mediating ethylene perception and signalling in tomato fruit, we analysed the expression of two ethylene receptor genes and five ethylene signalling-related genes. Both ethylene receptor genes, *SlNR* and *SlETR4* were up-regulated in M50-fruit by 2.3-fold (Fig. 5C). The *CTR* and *EIN* gene are involved directly in transducing the ethylene receptor signal (Seymour *et al.*, 2013). *SlEIN*/*EIL1* and three genes were up-regulated by exogenous melatonin, whereas the expression of *SlCTR1* and *SlEIN*/*EIL2* was almost unresponsive to melatonin treatment (Fig. 5C). Ethylene response factor (ERF) is a well-known transcriptional regulator involved in controlling ethylene responses (Seymour *et al.*, 2013). In our study, *SlERF2* was highly up-regulated by 2.5-fold under melatonin treatment (Fig. 5C).

In this study, we observed that the climacteric peak of melatonin-treated samples occurred earlier, and ethylene production was increased compared with CK (Fig. 5A). Furthermore, at the climacteric peak time under 50 µM melatonin treatment, the expression of ethylene biosynthesis-related gene (*SlACS4*), ethylene receptor genes (*SlNR* and *SlETR4*), and ethylene signalling-related genes (*SlEIL1*, *SlEIL3*, and *SlERF2*) were up-regulated to varying degrees. Taken together, these results indicate that melatonin may affect the ethylene production by control of gene expression on ethylene biosynthesis and signal transduction pathway.

### Melatonin contributed to tomato aroma and flavour

**Fig. 6.**
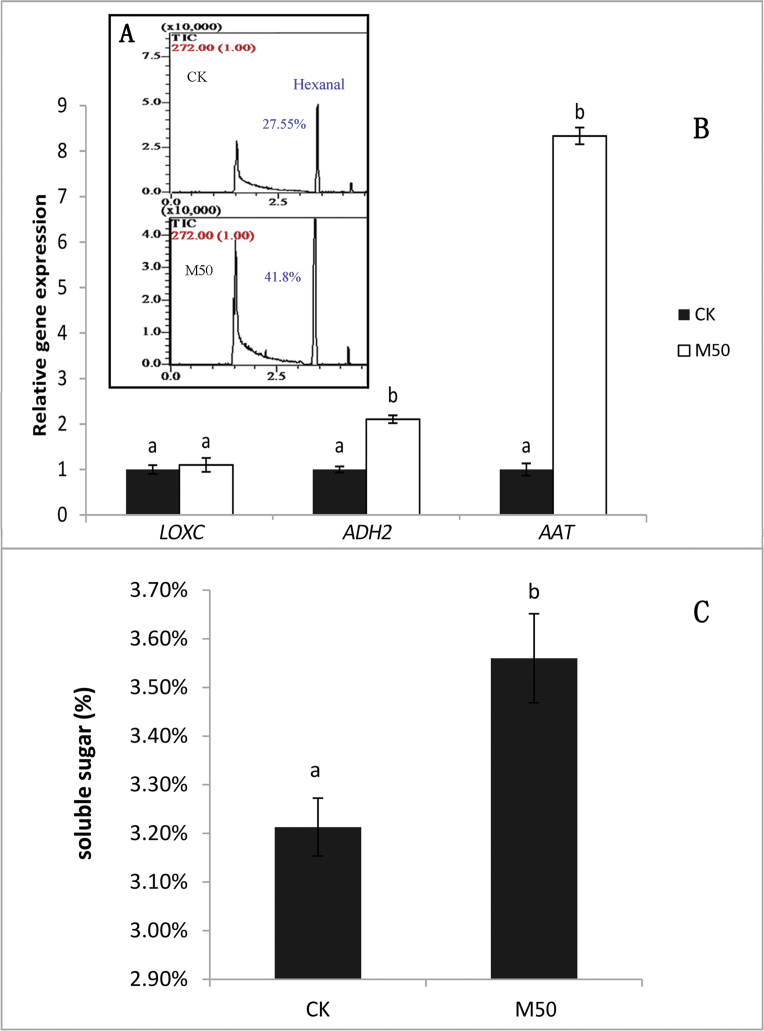
Melatonin has an effect on tomato flavour components. CK: samples pre-treated with water; M50: samples pre-treated with 50 μmol/L melatonin. (A) The volatiles content of hexanal, and (B) related gene expression. (C) Water-soluble sugar of tomato fruit at 17 d after treatment. Vertical bars at each time point represent the LSD when significant at *P*=0.05. (This figure is available in colour at *JXB* online.)

To understand the effect of melatonin on aroma in tomato fruit, we used the GC-MS technique to detect the volatiles of tomato fruits. Hexanal, a compound related to ripe aroma and tomato-like flavour (Baldwin *et al.*, 1998; Maul *et al.*, 2000), increased by 51.8% in melatonin-treated fruits (Fig. 6A).

Furthermore, we analysed the expression of *SlLoxC*, *SlADH2*, and *SlAAT*, which are involved in volatile compound (such as hexanal) metabolism. Our data showed that *SlADH2* and *SlAAT* were both up-regulated by melatonin treatment, whereas *SlLoxC* did not show a significant response (Fig. 6B). The expression level of *SlAAT* markedly increased by 8-fold under 50 µM melatonin treatment when compared with CK.

To better understand the role of melatonin in fruit ripening, we also measured soluble sugar in melatonin-treated and control fruits. Significantly higher levels of soluble sugar (by 10.9%) were detected in M50-fruit compared with control sample at 17 DAT (Fig. 6C).

Both the sugars and volatiles are critical to good flavour in tomato fruit (Klee and Giovannoni, 2011). Volatiles determine the unique flavour of tomato, whereas the sugars are essential for good taste. We inferred that the biosynthetic pathway of volatiles may have been affected by melatonin treatment. Moreover, the level of soluble sugar in M50-fruit was higher than that of CK, indicating that melatonin may have a critical role in tomato flavour.

## Discussion

Melatonin was detected in tomato in 1995 (Dubbels *et al.*, 1995), and its level in the pericarp of tomato fruit increases from mature green stage to red stage (Van Tassel *et al.*, 2001; Okazaki and Ezura, 2009). Our study found that exogenous melatonin could accelerate the process of tomato fruit postharvest ripening, as indicated by accelerated red colour development and increased fruit softening, water loss, pigment accumulation, and flavour formation. Moreover, the expression of associated genes was up-regulated by melatonin. The increment of ethylene production in melatonin-treated fruit is a consequence of up-regulated *SlACS4* expression. The expression of genes involved in ethylene signal transduction pathway such as *SlNR*, *SlETR4*, *SlEIL1*, and *SlEIL3* was altered by melatonin, suggesting that ethylene perception and sensitivity was affected by melatonin.

During ripening, tomato fruit colour changes from green to red as the result of carotenoid accumulation and chlorophyll degradation. Lycopene, the most abundant carotenoid found in tomatoes, is also important in human nutrition and health, as a precursor of Vitamin A and a potent antioxidant (DellaPenna and Pogson, 2006; Klee and Giovannoni, 2011). Lycopene biosynthesis in plants occurs via the central isoprenoid pathway beginning with the formation of phytoene from geranylgeranyl diphosphate (GGPP) and four desaturation steps (Li and Yuan, 2013). The first step of carotenoid biosynthesis is catalysed by PSY1, a fruit-specific phytoene synthase. *CRTISO* encodes carotenoid isomerase, an enzyme that isomerizes poly-*cis*-lycopene to all-*trans*-lycopene (Isaacson *et al.*, 2004). Many hormones can impact lycopene biosynthesis. Recently, advances have been made in the regulation mechanism of ethylene on lycopene biosynthesis in tomato fruit. The Nr mutant (a single amino acid change in Nr ethylene receptor) exhibits reduced ethylene sensitivity and accumulates low amounts of lycopene in ripened fruits (Lanahan *et al.*, 1994). Moreover, PSY is under strong positive ethylene control during ripening (Klee and Giovannoni, 2011). In our research, melatonin treatment enhanced lycopene accumulation (Fig. 1A) and ethylene production (Fig. 5A), implying that melatonin may increase the content of lycopene by impacting ethylene biosynthesis and signalling.

During fruit development and ripening, fruit softening, primarily owing to cell wall degradation and reduction of intercellular adhesion, is a prerequisite for a desirable eating quality of a fruit. Fruit cell walls are a complex network of polysaccharides and proteins, wherein the primary cell wall contains, on average, about 35% pectin, 25% cellulose, 20% hemicellulose, and 10% structural protein (Li *et al.*, 2010). Cell wall modifications lead to progressive degradation of cell wall polymers and loss of integrity of the middle lamella, which is rich in pectins that control cell-to-cell adhesion, thus influencing fruit texture (Brummell and Harpster, 2001). In general, pectins are the first cell wall components to be modified during fruit ripening. PE (pectin esterase) is the main pectin de-esterification enzyme, and PG (polygalacturonase) is the main enzyme promoting pectin solubilization. PEs may act synergistically with PGs, with PE action resulting in the generation of homogalacturonan (HG) substrates that are more susceptible to PG-mediated hydrolysis (Seymour *et al.*, 2013). Depolymerization of hemicelluloses is also a common feature of ripening fruit; endo-transglycosylase hydrolase (XTH) plays an important role in this process by modifying xyloglucan, the predominant hemicellulose of cell walls (Brummell, 2006). In tomato, both ethylene biosynthesis and signal transduction play important roles in regulating fruit ripening-related textural changes (Li *et al.*, 2010). Gene expression analysis has revealed that ethylene directly regulates the transcription of both a softening-related PpPG gene and an expansin (*PpExp3*), thus suggesting that the rapid loss of firmness in ripening fruit is caused by cell wall metabolism, and is controlled by ethylene at the transcriptional level (Hayama *et al.*, 2006). Moreover, *PpPG2* and *PpPME2* are reported to be involved in ethylene-regulated solubilization of polyuronides in peach fruit (Murayama *et al.*, 2009). In this study, we found that ethylene production and signalling was affected by melatonin, and M50-fruit was softer than CK-fruit (Fig. 3A). Our findings suggest that melatonin may influence ethylene to regulate fruit textural changes.

Water movement has been considered a possible contribution to fruit firmness, and aquaporin proteins may influence water loss during ripening (Seymour *et al.*, 2013). Aquaporins (AQPs) are water channel proteins that allow rapid and selective transport of water across membranes, which would presumably also affect water movement out of the cell. Fruit softening could be associated not only with cell wall disassembly, but also to loss of cell turgor mediated by water flow through aquaporins (Alleva *et al.*, 2010). Aquaporins are also considered to be regulated by ethylene during fruit ripening; for example, the stimulation of AQP2 by ethylene in grape skin tissues reached 300% (Chervin *et al.*, 2008). Some aquaporins investigated in our study were up-regulated in melatonin-treated fruit (Fig. 4B). This result was consistent with the advancement of ethylene production, implying that melatonin may have an effect on the ethylene pathway to enhance the expression of aquaporin genes, leading to increased water loss in melatonin-treated fruit.

At present, consumers are concerned about the quality of fruit, including nutritive value, aroma, colour, and shelf life. Colour and aroma volatiles are two major quality attributes of tomato fruits. AAT is capable of combining various alcohols and acyl CoAs, resulting in the synthesis of a wide range of esters. Aliphatic esters contribute to aroma of nearly all fruit and are emitted by vegetative tissues (Schwab *et al.*, 2008). ADH genes that are suspected to participate in the production of aromas are expressed in a developmentally regulated manner, particularly during fruit ripening (Manriquez *et al.*, 2006). Both *AAT* and *ADH2* are involved in the biosynthetic pathway leading to esters (such as hexanal) formation. Synthesis of the volatiles that are up-regulated during ripening is dependent upon ethylene, as there are no ripening-associated increases in the ethylene-insensitive Nr mutant (Zhu *et al.*, 2005; Klee and Giovannoni, 2011). Ethylene also affects the essential enzymes involved in the volatile biosynthetic pathways to influence the level of volatiles, such as lipoxygenase (LOX), alcohol dehydrogenase (ADH), and alcohol acyltransferase (AAT) (Zhu *et al.*, 2005). Melatonin treatment up-regulated *ADH2* and *AAT* gene expression (Fig. 6B) and influenced ethylene biosynthesis, ethylene perception, and ethylene signalling. Therefore, melatonin may have a role in ethylene biosynthesis and signalling to indirectly influence the level of volatiles.

**Fig. 7.**
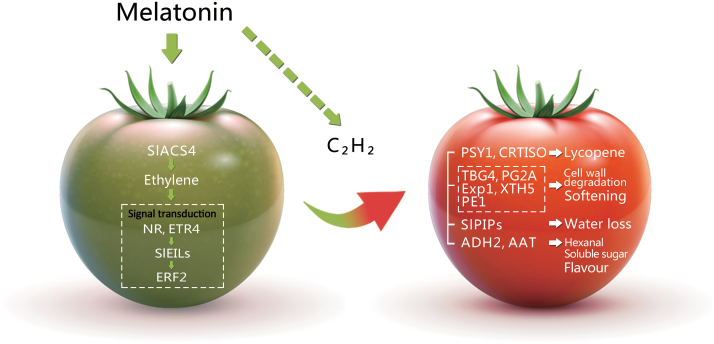
A model of the role of melatonin in postharvest ripening tomato fruits. Melatonin may promote tomato fruit ripening by affecting ethylene production and signalling. Arrows represent positive regulation, the dotted lines represent regulatory steps in which a direct physical link between upstream and downstream components has yet to be demonstrated.

Ethylene plays a key regulatory role during ripening of many fruits by regulating carotenoid lycopene synthesis, conversion of starch to sugars, and enhancing cell wall-degrading enzyme activity. Exogenous melatonin could promote tomato fruit ripening during postharvest life, thus positively impacting ethylene production and signalling. We can infer that melatonin-induced fruit ripening events, such as lycopene accumulation, cell wall degradation and volatiles biosynthesis, are dependent on ethylene biosynthesis and signal transduction (Fig. 7). The findings expanded our understanding of melatonin function in ripening tomato fruits, providing evidence that melatonin is involved in fruit maturation.

## Supplementary data

Supplementary data are available at *JXB* online.

Table S1. The primers used for qRT-PCR.

## Author contributions

Q. Sun, N. Zhang, B. Zhao and Y.-D. Guo designed the research; Q. Sun, N. Zhang, J. Wang, H. Zhang, D. Li, J. Shi, R. Li performed the research; Q. Sun, N. Zhang, S. Weeda, S. Ren, and Y.-D. Guo analysed data; Q. Sun,S. Weeda, S. Ren, and Y.-D. Guo wrote the paper.

## Supplementary Material

Supplementary Data
