# Supplementary material for: Melatonin promotes ripening and improves quality of tomato fruit during postharvest life
Source: J Exp Bot. 2014 Aug 21;66(3):657–68. doi: 10.1093/jxb/eru332 (PMC4321535; doi:10.1093/jxb/eru332)
Supplement: Supplementary Data [file supp_66_3_657__index.html]

Melatonin promotes ripening and improves quality of tomato fruit during postharvest life — Melatonin promotes ripening and improves quality of tomato fruit during postharvest life — Supplementary Data 

# Melatonin promotes ripening and improves quality of tomato fruit during postharvest life

## Supplementary Data

Data files

**Files in this Data Supplement:**

- Supplementary Data - Supplementary Data
