# Supplementary material for: Melatonin promotes ripening and improves quality of tomato fruit during postharvest life
Source: J Exp Bot. 2014 Aug 21;66(3):657–68. doi: 10.1093/jxb/eru332 (PMC4321535; doi:10.1093/jxb/eru332)
Supplement: Supplementary Data [file supp_eru332_jexbot129056_file001.pdf]

Table S1. The primers used for qRT-PCR.

Gene ID	Gene names	Primers
M84744.1	PSY1-S	TTCAGAGTGGCCATTGTTGAAAGA
	PSY1-A	ACAACCCATAACAAGGCAACAG
AF416727.1	CRTISO-S	GCTTTGTGGCTTGAGTTGGG
	CRTISO-A	GCTGCCACCAATCCACCTAT
X04583.1	PG2A-S	AGGCTTTGGATTGCTTTTGA
	PG2A-A	AGAAGGTTAAGGCCGTTGGT
AY497475.1	XTH5-S	GGATGCTTCAGATTGGGCTA
	XTH5-A	CAACTTGTTCCAATGGGTCC
AF548376.1	EXP1-S	TTTTACGGCGGAAGTGATGC
	EXP1-A	AGTGAGGGCGAGGAGGGTTA
AF020390.2	TBG4-S	CTCAAAAGCAGCCCCTGACT
	TBG4-A	CTGAGAAGGTTGTCCGCAGT
X74638.1	PE1-S	GGGACGGAGATTTTGCGTTG
	PE1-A	TGTTATGCTTGCTCTGCCCT
Solyc08g081190.2.1	PIP12-S	CTATCATCTACAACGACGAGCA
	PIP12-A	CATTGAAGGAGAAACTTGAACA
Solyc02g083510.2.1	PIPQ-S	ATACCCAACGTGTAGCATCACTCTC
	PIPQ-A	CCAGCAGTGGAATACACGAGAACA
Solyc10g084120.1.1	PIQ21Q-S	ATTGACCCTGAGGAACTTGGAAAA
	PIP21Q-A	TCACCATCACTTTGGCTCTTGTAG
Solyc11g069430.1.1	PIP22Q-S	TACTCCGCAAAGGATTACACTGAT
	PIP22Q-A	AGCCCAAGCAATACCAAGTAAACC
Solyc05g055990.2.1	4Q-S	GTCCTCTTCCAGCCATCCA
	4Q-A	ACCACTGAGCACAATGTTACCG
NM_001247249.1	ACS2-S	AAAGCGCGATGAGGTTAGGT
	ACS2-A	CAACACCTACGAACCTCCGAA
M63490.1	ACS4-S	AGATCGCACTTGCAAGGATTC
	ACS4-A	ATTACCTATGTTGGGCCCGTG
AF532976.1	ACO1-S	CAAACAGACGGGACACGAAT
	ACO1-A	ATTGGATCACTTTCCATTGCCT
U38666.1	NR-S	ATCGCATTCTCCGTCGTCAT
	NR-A	TCCATTATCTCGTTTCGTCCC
AF118843.1	ETR4-S	GTTCTTGGGCTTCAACTGCG
	ETR4-A	ACAGCAGGGCTAAGAACACC
AF328784.1	EIL1-S	GCACGAGCTGAGTTCCAGT
	EIL1-A	CGGAGCAGACACTTCCACTT
AF328785.1	EIL2-S	GAAACCCCTTTGAGGTTGCT
	EIL2-A	GGCGTTGTTTGACAGCATCA

AF328786.1	EIL3-S	CACGAGCCTCTCTTCTTCTTAC
	EIL3-A	ACCGGCTTATGCTCAACTTC
AY192368.1	ERF2-S	CGTTTGTCATCCACCGACCT
	ERF2-A	GTCACGAATTTCAGCAGCCC
AY079048.1	CTR1-S	GCCTGGAGTTTTAAGCGAACC
	CTR1-A	GCAGCTCCAGCTGAAGAATCA
AY534531.1	AAT-S	TCAATAAATTACCACAAGCCAAAAC
	AAT-A	GGCCCTTCAATGAGTCTACCA
M86724.1	ADH2-S	ATGTGTCCATGATGGCTGGG
	ADH2-A	GGTGATGATGCAACGAAGGC
U37839.1	LoxC-S	CAAATGAACCACATGGCTTG
	LoxC-A	TGGCCAGAAGTTACCCAAAC
SGN-U580609	Actin-S	TGTCCCTATCTACGAGGGTTATGC
	Actin-A	AGTTAAATCACGACCAGCAAGAT
